# Mebendazole as a Candidate for Drug Repurposing in Oncology: An Extensive Review of Current Literature

**DOI:** 10.3390/cancers11091284

**Published:** 2019-08-31

**Authors:** Andrea Emanuele Guerini, Luca Triggiani, Marta Maddalo, Marco Lorenzo Bonù, Francesco Frassine, Anna Baiguini, Alessandro Alghisi, Davide Tomasini, Paolo Borghetti, Nadia Pasinetti, Roberto Bresciani, Stefano Maria Magrini, Michela Buglione

**Affiliations:** 1 Department of Radiation Oncology, Brescia University, 25123 Brescia, Italy; 2 Department of Radiation Oncology, ASST Spedali Civili of Brescia, P.le Spedali Civili 1, 25123 Brescia, Italy; 3 Radiation Oncology Service, ASST Valcamonica, 25040 Esine, Italy; 4 Department of Molecular and Translational Medicine, Unit of Biotechnology, University of Brescia, Viale Europa 11, 25123 Brescia, Italy

**Keywords:** chemoresistance, radioresistance, mebendazole, repurposing, cancer stem cells, cancer, CSC, anticancer, stemness

## Abstract

Anticancer treatment efficacy is limited by the development of refractory tumor cells characterized by increased expression and activity of mechanisms promoting survival, proliferation, and metastatic spread. The present review summarizes the current literature regarding the use of the anthelmintic mebendazole (MBZ) as a repurposed drug in oncology with a focus on cells resistant to approved therapies, including so called “cancer stem cells”. Mebendazole meets many of the characteristics desirable for a repurposed drug: good and proven toxicity profile, pharmacokinetics allowing to reach therapeutic concentrations at disease site, ease of administration and low price. Several in vitro studies suggest that MBZ inhibits a wide range of factors involved in tumor progression such as tubulin polymerization, angiogenesis, pro-survival pathways, matrix metalloproteinases, and multi-drug resistance protein transporters. Mebendazole not only exhibits direct cytotoxic activity, but also synergizes with ionizing radiations and different chemotherapeutic agents and stimulates antitumoral immune response. In vivo, MBZ treatment as a single agent or in combination with chemotherapy led to the reduction or complete arrest of tumor growth, marked decrease of metastatic spread, and improvement of survival. Further investigations are warranted to confirm the clinical anti-neoplastic activity of MBZ and its safety in combination with other drugs in a clinical setting.

## 1. Introduction

The main limit of chemotherapy and radiotherapy is the development of refractory clones resistant to antineoplastic agents and prone to local and metastatic spread [1]. Targeted therapy and immunotherapy only partially overcome radio and chemo-resistance and they can lead to the development of refractory clones [2]. Furthermore, their use is limited to selected patients and is too expensive for the majority of healthcare systems worldwide [3]. Cancer cell survival is mediated by many molecular mechanisms including overexpression of oncogenes activating pro-survival pathways, mutation of tumor suppressors genes, uncontrolled activity of DNA repair enzymes, expression of transporter pumps mediating drug efflux, autophagy, and unregulated angiogenesis [4,5]. Cancer progression is not determined only by intrinsic neoplastic cells features, but is also controlled by interaction with the tumor micro-environment: cancer-associated fibroblasts are responsible for paracrine secretion of growth factors [6], endothelial cells mediate angiogenesis, while the extracellular matrix might limit drug availability and stimulate invasion and migration [7]. Abnormal activity of the immune response is also essential in tumor progression: M2-polarized macrophages, regulatory T-cells, and myeloid-derived stromal cells promote an immunotolerant state and also cancer cells can directly express immunosuppressive cytokines [8,9]. A crucial role in disease persistence and progression is played by the so called “cancer stem cells” (CSCs), characterized by tumor-initiating capacity, self-renewal, and enrichment in the survival mechanisms listed above [10]. At present, there are no drugs approved to specifically target this cell population. Drug development is burdened by elevated costs (approximately $1–2.5 billions [11] to discover a candidate compound and take it through all the trials needed for FDA approval) that limit the pace of new molecules discovery. Drug repurposing, the identification of novel indications for existing and already approved compounds, is becoming attractive as a mean to minimize the chance of failure due to the adverse effects, mitigate the economic load, and accelerate the approval process [12]. The ideal candidate for drug repurposing is a molecule with good and proven toxicity profile, pre-clinical evidence of efficacy, pharmacokinetics allowing to reach therapeutic concentrations at disease site, synergy with approved anticancer treatments and low price. Since mebendazole (MBZ), a broad-spectrum anthelmintic drug, seems to fulfill all these characteristics, we performed an extensive review of the current literature (last update 13 August 2019) regarding its use as an investigational drug for anticancer treatment.

## 2. Current Indications and Safety

**Figure 1:**
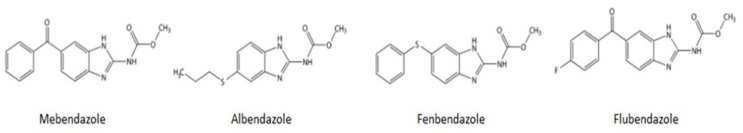
Chemical structures of benzimidazole anthelmintics commonly prescribed for human (mebendazole and Albendazole) and veterinary (Fenbendazole and Flubendazole) use.

Mebendazole is a synthetic benzimidazole effective against a broad spectrum of intestinal helminthiasis. Chemical structures of MBZ and other benzimidazole anthelmintics commonly prescribed for human (Albendazole, ABZ) and veterinary (Fenbendazole, FBZ, and Flubendazole) use are shown in Figure 1. The most common formulation of MBZ is as 100–500 mg tablets. The dosage varies according to the type of infection: 100 mg single dose for pinworms, 100 mg bis in die (b.i.d.) for three days (d) for roundworms or hookworms. Mebendazole has also been prescribed at high doses on a long-term basis for the treatment of echinococcosis: the WHO guidelines [13] recommend 40–50 mg/kg/day for at least 3–6 months and at least two years in case of alveolar echinococcosis. For this indication, MBZ has been prescribed also at higher doses both in adults [14] and in children [15]. The safety of MBZ has been evaluated in 6276 subjects in 39 clinical trials and in decades of post-marketing experience [16]. At the usual low-dose regimens, side effects are limited to abdominal pain and discomfort, flatulence, and diarrhea. During high-dose treatment rare instances of neutropenia and marrow aplasia have been reported, which were almost always reversible with complete recovery after a few days of drug suspension and happened more frequently in patients with altered drug metabolism [17]. High doses have also been linked to rare reports of alopecia, allergic reaction, and elevations in transaminases levels with isolated cases of reversible liver injury due to the fact of hypersensitivity [18].

## 3. Pharmacokinetics and Pharmacodynamics

Mebendazole has poor bioavailability: following oral administration, approximately 17–20% of the dose reaches the systemic circulation [19,20] due to the fact of incomplete absorption and extensive first-pass effect. Great inter-individual variation in pharmacokinetics have been reported [21,22,23,24,25] (Table 1) after administration for helminthic infections and in healthy volunteers. Chronic therapy resulted in greater Cmax (maximum serum concentration) and AUC compared to single administration, suggesting some degree of enterohepatic circulation [21]. Systemic bioavailability of MBZ is also enhanced by concomitant intake of fatty foods [22,24]. Mebendazole is 90–95% protein-bound in plasma, clearance is predominantly as metabolites in bile and feces, and only 2–5% is excreted in urine [25]. Lipophilic properties and low molecular weight allow MBZ to pass through the blood–brain barrier [26] (BBB) and this is clinically confirmed by the efficacy of the drug in the treatment of cerebral echinococcosis. In vivo, pharmacokinetic analysis of the three polymorphs of MBZ [27] (A, B, and C) showed that, after oral administration of 50 mg/kg to mice, B and C had good BBB penetration, with a peak concentration of 7.1 μM in brain tissue. Mebendazole acts by binding to the colchicine-sensitive site of β-subunit of helminthic tubulin, hindering the dimerization with α-tubulin [28] and, thus, compromising cellular structures, intracellular transport, and glucose absorption.

## 4. Preclinical Evidence of Anticancer Activity

**Figure 2:**
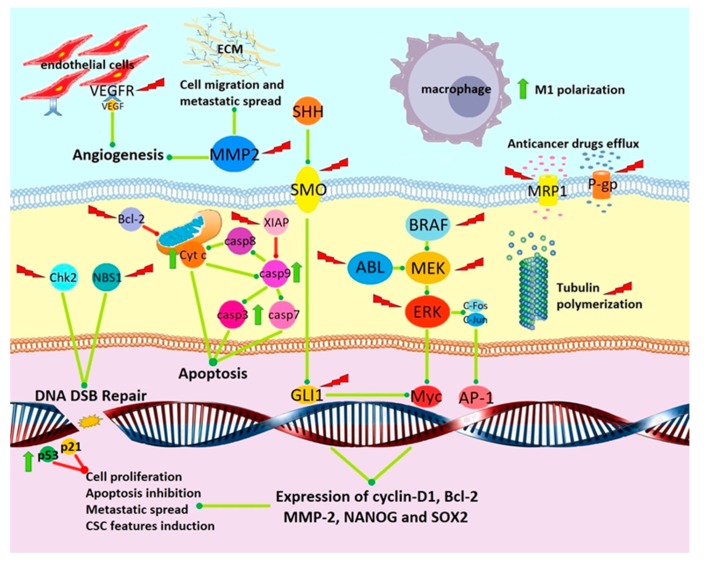
Schematization of antitumoral effects of MBZ. Green lines = induction and/or activation; red lines = inhibition and/or downregulation; green arrow = induction/overexpression induced by MBZ; red lightning bolt = inhibition/downregulation/degradation induced by MBZ. ECM = extracellular matrix; Cyt c = cytochrome c; casp3-7-8-9 = caspase 3-7-8-9; CSCs = cancer stem cells.

Preclinical activity of MBZ against cancer is summarized in Figure 2. Studies regarding the in vitro and in vivo anticancer effect of MBZ are sorted according to mechanism of action.

### 4.1. Tubulin Depolymerization

Mebendazole was firstly tested against cancer in 2002 [29]: exposure of human NSCLC cell lines to the drug-induced tubulin depolymerization, resulting in mitotic arrest. Expression of p53 and p21 proteins was induced after 24 h and after 48 h cell apoptosis (mediated by both cytochrome c and caspases) was reported. The in vivo antitumor effect of MBZ was then examined in H460 mice xenografts: significant suppression of tumor growth was observed by 14 days after treatment start. A relevant fraction of apoptotic cells was detected in samples from MBZ group mice. Bai et al. [30] tested the activity of MBZ on glioblastoma (GBM) cell lines in vitro and in vivo. Mebendazole significantly inhibited tubulin polymerization in 060919 cells, a human stem-like line resistant to temozolomide (TMZ). The antiproliferative efficacy of MBZ and TMZ on GBM cell lines was tested in vitro: the half maximal inhibitory concentrations (IC_50_) of MBZ appeared to be in a close range (0.11–0.31 µM), while TMZ showed IC_50_ ranging from 8.7 to 547 µM. Subsequently, a GL261 syngenic intracranial mouse glioma model was established and MBZ was administered alone or in combination with TMZ. Mean survival was 29 d in the control group, 41 d in the TMZ group, and 50 d in the TMZ + MBZ group, which was not significantly different from the survival increase with MBZ alone. Albendazole showed a less evident increase in survival (20–30%) at both dosages tested. Mebendazole was then tested in a mouse intracranial 060919 xenograft model, resulting in survival increase (65 d versus 48 d) compared to the control, while TMZ or ABZ had no effect. The measurement of luciferase activity confirmed that tumor growth was inhibited by MBZ treatment in both models. The use of the anti-tubulin agent vincristine (VCR) is limited by its toxicity and poor BBB penetrance. De Witt et al. [31] proposed MBZ as a replacement for VCR in the treatment of brain tumors in view of its favorable pharmacokinetic and toxicity profile. In vitro, cell toxicity of MBZ on GL261 murine glioma was mediated by inhibition of microtubule formation: the concentration required to achieve a half-maximal effect (EC_50_) of MBZ for microtubule depolymerization (132 nM) was similar to that for viability suppression and mitotic arrest induction. Moreover, treatment with polymorph C MBZ showed a significant increase in survival in an intracranial GL261 murine allograft, whereas VCR failed to show any efficacy. Pinto et al. [32] tested MBZ cytotoxic activity against gastric cancer cell lines obtained from patients. Mebendazole disrupted the microtubule structure and significantly inhibited invasion and migration at concentrations as low as 0.1 μM. Activity of MMP-2 significantly decreased at all tested concentrations. Mebendazole was more potent as an antiproliferative agent against gastric cancer cell lines (IC50 0.39–1.25 μM) than several other clinically approved chemotherapic agents tested such as 5-fluorouracil (5-FU), oxaliplatin, gemcitabine, irinotecan, cisplatin, paclitaxel, and doxorubicin. The association between MBZ and 5-FU increased the cytotoxicity in a non-statistically significant manner when compared to each drug alone.

### 4.2. Angiogenesis Inhibition

Mukhopadhyay et al. demonstrated the antiproliferative effect of MBZ on human NSCLC cell lines (A549, H129, and H460) reporting an IC_50_ of ~0.16 μM, while no effect was shown on normal human umbilical vein endothelial cells (HUVECs) or fibroblasts [33]. Mebendazole also profoundly inhibited the growth of breast, ovary, and colon carcinoma and osteosarcoma cell lines, producing IC_50s_ from 0.1 to 0.8 μM. The in vivo response to MBZ was then tested in an H460 xenograft model: growth inhibition was dose dependent and 1 mg every other day (e.o.d.) almost completely arrested tumor growth. The experiment was repeated in a K1735 murine melanoma allograft, reporting a growth inhibition of ~70%. Mice treated with MBZ showed no sign of toxicity. A significant decrease in the number of CD31+ endothelial cells and a 75% decrease in hemoglobin content was observed in tumor samples from MBZ-treated xenografts, demonstrating a reduction of neovascularization. Angiogenesis was further evaluated in vivo using the dorsal air sac method: both the number and caliber of the blood vessels were significantly reduced in treated mice. In an A549 xenograft model, MBZ was also able to inhibit the formation of lung metastatic colonies by about 80% without any apparent toxicity, while treatment with the established tubulin-inhibitor paclitaxel had no effect on metastasis formation. Medulloblastoma is the most common malignant brain tumor in children. Four molecular groups [34], including group 2 driven by sonic hedgehog (SHH) and group 3 associated with MYC amplification, have been identified. Bai et al. tested MBZ on a panel of eight medulloblastoma cell lines [35], obtaining IC_50s_ for cell growth of 0.13–1 μM. Mebendazole also inhibited vascular endothelial growth factor receptor 2 (*VEGFR2*) autophosphorylation at 1–10 μM in cultured HUVECs and with an IC_50_ of 4.3 μM in a cell-free kinase assay. Mebendazole was then tested on two murine allograft models obtained by intracranial injection of medulloblastoma cell lines bearing SHH pathway mutations. Mebendazole improved the survival of the mice up to 150% and luminescence images confirmed growth inhibition. Mebendazole activity was also demonstrated in an orthotopic intracranial model of human D425 c-MYC amplified medulloblastoma. Compared to the control, survival was prolonged from 21 to 48 d and tumor burden reduction was again displayed, as reflected by the luciferase signals. Analysis of tumor sections from treated mice revealed significant reduction of tumor angiogenesis, while the microvessel density in the normal brain parenchyma was not affected [35]. Williamson and investigators from the previous group evaluated the effect of MBZ on colorectal cancer in vitro and in vivo [36]. In vitro, the IC_50S_ for proliferation in human colorectal cell lines were 0.20–0.81 μM. The efficacy in vivo was then tested on HT29 or SW480 adenocarcinoma xenografts, with a volume and weight reduction of respectively 62% and 65% in HT29 and 67% and 59% in SW480 models. Treatment also led to a significant reduction of Ki67 expression in tumor samples. The chemopreventive activity of MBZ alone or in combination with the nonsteroidal anti-inflammatory sulindac in the ApcMin/+ mouse model of familial adenomatous polyposis was then assessed. Mebendazole reduced the number of tumors by 56% as a single agent and up to 90% in combination with sulindac. Mebendazole impaired tumor angiogenesis and inhibited *VEGFR2* kinase activity, microvessel density was reduced by 51% in samples from ApcMin/+ polyps. The expression of proteins critical for adenoma initiation and progression (e.g., *MYC*, *COX2*, and *Bcl-2*) and pro-inflammatory cytokines and growth factors (*TNF*, *IL6*, *VEGF*, *IL1β*, *G-CSF*, *GM-CSF*, *FGF2*) was reduced in tumor samples after MBZ or combination treatment.

Sung et al. [37] demonstrated the anti-angiogenic activity of MBZ on HUVECs: proliferation and *VEGF*- or *bFGF*-induced migration and tube formation were inhibited in a dose-dependent manner in several culture conditions; in contrast, FAK and ERK1/2 phosphorylation induced by VEGF or bFGF was not affected. Mebendazole also led to p53 accumulation, arrest in G2-M phase, and, consequently, apoptosis in a time- and dose-dependent manner. A marked induction of autophagy by MBZ was also noted and addition of autophagy inhibitors resulted in marked enhancement of anti-proliferative and pro-apoptotic effects of MBZ.

### 4.3. Inhibition of Signal Transduction Pathways Involved in Cancer Progression

The SHH signaling pathway, involving downstream effectors smoothened (SMO) and glioma-associated homolog 1 (GLI1), is constitutively activated in many types of cancer [38]. A study published in 2015 by Larsen et al. demonstrated the efficacy of MBZ as a hedgehog inhibitor on human medulloblastoma cell line DAOY [39]. In vitro MBZ inhibited SMO mutant proteins that confer vismodegib resistance, and a combination of MBZ and vismodegib achieved additive inhibition of canonical SHH signaling. Mebendazole inhibited GLI1 expression with an IC_50_ of 516 nM. Cell proliferation was inhibited at a concentration as low as 100 nM and viability was significantly impaired at 1 μM. Mebendazole also inhibited the assembly of the primary cilium, a tubulin-based structure with a central role in SHH and other pathways involved in carcinogenesis. The activity of MBZ in vivo was then assessed in a DAOY intracranial mouse xenograft. Treatment significantly increased survival from 75 d in the control group to 94 d in the group administered MBZ 25 mg/kg and 113 d in the 50 mg/kg group. Bioluminescence imaging demonstrated a marked reduction of tumor cell proliferation, while levels of GLI1 and PTCH2 transcripts were reduced in cells from tumor samples. The X-linked inhibitor of apoptosis (*XIAP*) is a protein able to effectively prevent cell death by inhibition of caspase 3, 7, and 9. Its expression has also been shown to increase with progressive disease stage in melanoma [40]. Doudican et al. evaluated the effect of MBZ on M-14 and A-375 human melanoma cell lines [41]. Treatment resulted in a time-dependent decrease in *XIAP* levels, which inversely correlated with an increase in apoptosis markers cleaved PARP and caspase 9. To confirm the antitumor effect of MBZ in vivo, a M-14 xenograft model was established: MBZ inhibited tumor growth comparably at both doses tested (83% and 77% inhibition, respectively) and was as effective as TMZ, without any toxicity. Increased Bcl2 phosphorylation, decreased levels of XIAP and enhanced cleavage of caspases 3 and 9 were detected in tissue samples of tumors from treated mice. Mutations of *BRAF* are found in about 65% of melanomas, while about 20% carry *NRAS* mutations [42]. Using computational methods coupled with kinase assays, Simbulan-Rosenthal et al. found that MBZ inhibits kinases involved in cancer pathways in the nanomolar range [43], especially BRAF and MEK. Patient-derived NRAS mutated (BAK and BUL) and BRAF mutated (STU) melanoma cell lines were exposed to increasing concentrations of the MEK1/2 inhibitor trametinib (T), MBZ or a combination of T + MBZ. Mebendazole showed weak cytotoxic activity as a single agent but synergized strongly with trametinib in BAK and BUL cell lines. Both trametinib and MBZ inhibited the MAPK/ERK pathway, downregulated its downstream targets, and induced markers of apoptosis. The effect of the combination of MBZ + T was more evident and happened earlier than after single drug treatment. The antitumor effect of MBZ and T was also tested in vivo: BAK xenografts were treated with low- or high-dose T, MBZ or a combination of the drugs. Trametinib and MBZ as single agents did not show significant antiproliferative effect, while tumor growth was significantly inhibited in both groups receiving combinations of the drugs. Analysis of tumor specimens showed that MEK1/2 and ERK1/2 phosphorylation was reduced by both drugs alone and completely abrogated only by the combination of the two drugs. The transcription factor *c-MYB* plays a central role in the development of acute myeloid leukemia (AML) and other tumors [44]. Walf-Vorderwülbecke et al. used a *c-MYB* gene expression signature from AML cells to probe a library containing over 1,500,000 gene expression profiles and identified MBZ as the most efficient *c-MYB* targeting drug [45]. Mebendazole was further investigated against eight different AML cell lines in vitro: IC_50s_ for cell viability ranged between 0.07 and 0.26 µM. Mebendazole treatment inhibited *c-MYB* protein expression in all cell lines examined and also induced *c-MYB* degradation via the proteasome. Mebendazole resulted in a more than 80% reduction in colony formation on THP1 AML cells, but no effect was observed on normal CD34+ cord blood cells. In vivo MBZ showed activity in a luciferase expressing THP1 AML murine xenograft determining inhibition of leukemia progression (assessed by bioluminescence imaging) and prolonged survival of treated mice compared to the control. Multi-drug resistance proteins play an essential role in chemoresistance to several anticancer agents [46]. The effect of MBZ on the expression of those proteins was tested by Pinto et al. [47] on an intestinal type adenocarcinoma cell line, resulting in a significant decrease of mRNA and protein expression levels of P-glycoprotein1 (P-gp) and other MDR proteins.

The effect of MBZ and flubendazole (FBZ) on migration and proliferation of PE/CA-PJ15 and H376 oral squamous carcinoma cells and premalignant oral keratinocytes DOK was tested by Kralova et al. [48]. The IC_50_ values for proliferation inhibition were similar for MBZ (0.24–0.25 μM) and FBZ (0.19–0.26 μM), while normal oral keratinocytes and gingival fibroblasts were less sensitive to the treatment. Low concentrations of MBZ and FBZ led to inhibition of kinases (FAK) and GTPases (Rho-A, Rac1) involved in cell motility and metastatic spread and hindered cell migration of cancer cell lines at low concentrations, that had no effect on the normal gingival fibroblasts. Transforming growth factor beta (TGF-β) induced N-cadherin expression and promoted cellular motility in DOK cells and those effects were inhibited by both drugs even at very low concentrations (50 nM), while E-cadherin levels decreased after exposure to higher concentrations.

### 4.4. Sensitization to Chemotherapy and Radiotherapy

Recent studies suggest that microtubule inhibitors synergize with ionizing radiations (IRs) not only during mitosis, but also during interphase [49] by interfering with the trafficking of DNA damage response proteins (DDRp). Markowitz et al. combined IR with MBZ to treat human patient derived GBM14 glioblastoma and murine GL261 glioma cells [50]. In a viability assay, cells were treated with 25–150 nM MBZ either pre-IR or post-IR (when the mitotic index was 0%) and irradiated with 3–9 Gy. The radio-sensitizing effect of MBZ and vincristine (VCR) was quantified using the dose enhancement factor at the point of 50% (DEF_50_). In GL261 cells, DEF_50_ for MBZ ranged from 1.2 to 1.41, while in GBM14 cells, DEF_50_ ranged from 1.33 to 1.69. An identical set of experiments was performed with VCR in GL261 cells, with similar DEF_50s_ (1.34–1.53). Mebendazole hindered DNA repair through inhibition of the trafficking of DDRp from the cytoplasm to the nucleus. Two proteins mediators of DSB repair, Chk2 and Nbs1, were evaluated: in GL261 cells the EC_50_ for cytoplasmic sequestration of Chk2 (31 nM) and Nbs1 (25 nM) was very similar to the EC_50_ for radiosensitization (35 nM) and, surprisingly, lower than the EC_50_ for microtubule depolymerization and mitotic arrest. Treatment with MBZ also led to more sustained levels of γH2AX, a marker of DNA damage, in response to IR. A similar relation of the EC_50s_ was reported for VCR, further supporting the notion that the radiosensitizing effect takes place also during interphase and can be obtained at low concentrations. Zhang et al. performed a high-throughput screen to identify drugs that prevented radiation-induced conversion of triple-negative breast cancer (TNBC) cells into CSCs [51]. Several drugs that belong to the anthelmintic group (including FBZ, Tiabendazole, and Niclosamide) were identified and, among them, MBZ was chosen for further investigations. In vitro, MBZ was tested on human breast cancer cells representing luminal ER+ subtype (MCF7 and T47D) and TNBC (basal MDA-MB-231 and claudin-low SUM159PT). Exposure to a single fraction of IR induced de-differentiation in a dose-dependent manner in all cell lines, but the effect was more marked on TNBC cells. Mebendazole induced the arrest of both TNBC cell lines in the G2/M phase of the cell cycle and had significant radiosensitizing effect at all radiation doses tested. Moreover, it dose-dependently decreased the fraction of ALDH1 positive cells, resulting in a significant depletion of CSCs capable of self-renewal and inhibited the hedgehog survival pathway. Mebendazole also showed cytotoxic effect on all cell lines tested, increased the fraction of apoptotic cells both as a single agent and in combination with IR and caused a significant increase in DNA double-strand breaks. In vivo, MBZ was evaluated on SUM159PT mice xenografts: MBZ alone resulted in modest delay of tumor growth, while the effect of a single fraction of radiotherapy (10 Gy) was evident and was enhanced by the addition of MBZ. Coyne et al. analyzed the effect of MBZ against HER2+ human mammary adenocarcinoma SKBr-3 [52]: cytotoxicity was dose- and time-dependent, reporting an IC_50_ of about 0.35 and 0.25 μM after 96 or 182 h. Mebendazole also had a synergistic antineoplastic effect with immunochemotherapeutics obtained by covalent binding of anti-HER2 and anthracyclines or gemcitabine. Kipper et al. [53] tested MBZ, TMZ, and vinblastine (VBL) in vitro on several glioma cell lines at concentrations in the range of those measured in the plasma of patients treated with the usual therapeutic dose. Cell lines only partially sensitive to TMZ alone were entirely ablated by combining TMZ with VBL or MBZ, while the triplet was more effective on TMZ-tolerant cell lines. Meningioma is a usually benign tumor of the central nervous system, but approximately 5% of the cases are represented by a malignant variant characterized by dismal prognosis [54]. Skibinski et al. described the antitumoral effect of MBZ on malignant meningioma combined with IR [55]. In vitro, MBZ showed antiproliferative effects on several meningioma lines with IC_50s_ in the 0.26–0.42 μM range. Mebendazole induced cytotoxicity, increased levels of cleaved caspase-3 and PARP and reduced colony formation as a single agent and, to a greater extent, in combination with IR. A mice model was then obtained by intracranial implant of KT21MG1 human meningioma cells and xenografts were treated with local radiation therapy using a single dose (12 Gy) with or without MBZ. Both treatments improved median survival, significantly reduced tumor luminescence, and delayed tumor growth. Immunohistochemical staining revealed increased expression of cleaved caspase-3 and reduced levels of the angiogenesis marker CD31 in tumor samples from mice in the MBZ group, and, even more markedly, in combination with the treatment group. Zhang et al. tested the effect of MBZ on head and neck squamous cell carcinoma using two human cell lines, CAL27 and SCC15 [56]. Mebendazole exerted a more potent antiproliferative effect than cisplatin in vitro, with IC_50s_ of 1.28 and 2.64 μM, respectively, induced apoptosis as a single drug, and had a synergistic effect with cisplatin. Unexpectedly, MBZ increased the activity of some proliferation-related pathways in CAL27 cells, conversely to the inhibitory effect shown in SCC15. A xenograft model was established using CAL27 cells. Tumors from MBZ-treated mice were slightly larger, while histologic examination of tumor samples exhibited features suggestive of cell differentiation such as extensive keratinization and diminished expression of proliferation markers.

### 4.5. Induction of Apoptosis and Cytotoxicity

In a paper published in 2008, Martarelli et al. investigated the effect of MBZ on H295R, SW-13 (human adrenocortical cancer), and WI-38 (normal fibroblast) cells lines [57]. The two cancer cell lines showed dose-dependent growth arrest, with an IC_50_ of 0.23 μM for H295R and 0.27 μM for SW-13, while normal fibroblasts were not affected. Mebendazole also inhibited cell invasion in a dose-dependent manner, with an IC_50_ of 0.085 μM. Apoptosis mediated by cytochrome c, caspase-9, and caspase-3 was induced in H295R and SW-13 cells in a dose-dependent manner. In vivo, oral treatment of murine xenografts of H295R or SW-13 cells significantly inhibited tumor growth. Mebendazole reduced the tumor volume to a similar extent in both H295R (respectively ~50% and ~60% reduction) and SW-13 xenografts (respectively ~70% and ~60% reduction) compared to controls. The dose effect was more evident in a metastasis model originated by intraperitoneal injection of SW-13 cells, as 1 mg treatment reduced the mean number of metastases of ~50% while 2 mg lead to a reduction of ~75% compared to controls. Doudican et al assessed the in vitro activity of MBZ against melanoma [58]: a screening of 2000 compounds was performed using two chemoresistant cell lines (p53-mutant M-14 and p53-wild-type SK-Mel-19) and a melanocyte cell line. Ten molecules that inhibited the growth of melanoma cells yet were largely non-toxic to melanocytes were identified; of these, four were benzimidazoles and MBZ had the greatest inhibitory effect (IC_50_ 0.30–0.32 µM) compared to ABZ (0.7–1.2 Μm) and FBZ (1.2–1.4 µM). At higher concentrations, MBZ also induced apoptosis through phosphorylation of Bcl-2 and activation of both intrinsic and extrinsic mitochondrial pathways. During a pharmacokinetic study reported above [27], Bai et al. tested MBZ polymorphs on intracranial murine GL261 glioma allografts and human medulloblastoma D425 xenografts. Mebendazole-A showed low blood and brain concentrations and no antitumoral efficacy. In contrast, MBZ-B and MBZ-C both significantly improved mean survival both in syngenic GL261 and xenograft D425 murine models and their effect was potentiated by elacridar (a potent P-gp and ABCB1 inhibitor). In a recent study by Pinto et al. [59], MBZ significantly induced DNA double-strand breaks in AGP01 gastric cancer cells at all concentrations tested (0.1–1 µM) in the same extent of doxorubicin and paclitaxel, while, converse to the two established anticancer drugs, it did not cause any significant damage in human lymphocytes. Mebendazole also induced G2-M arrest followed by increased activity of caspase 3/7 and apoptosis in about 70% of treated cells. Protein expression and mRNA levels of the proto-oncogene c-Myc were both reduced by MBZ exposure.

### 4.6. Inhibition of Kinases

Dakshanamurthy et al. developed a computational proteochemometric method to predict potential drug–target interactions and identify compounds that could be repurposed for anticancer therapy [60]. A screening performed on 3671 FDA approved drugs identified MBZ as a potential inhibitor of VEGFR2. In vitro studies confirmed this finding: MBZ bound directly to VEGFR2 and affected its kinase activity with an IC_50_ value of 3.6 μM. In the HUVEC functional assay MBZ inhibited angiogenesis with an IC_50_ of 8.8 μM. The same group later demonstrated, using kinase binding assays, that MBZ was able to inhibit several kinases at nano- and micromolar concentrations [61]. TNF receptor-associated factor 2 (TRAF2) and NCK-interacting kinase—TNIK—is an activator of TCF4/β-catenin transcriptional program [62]. Tan et al. performed a screening on 1448 FDA-approved and currently marketed drugs [63]. Mebendazole was identified as a potent TNIK inhibitor and this finding was confirmed in tests performed by a patented screening platform. Mebendazole was able to selectively inhibit 91.8% of TNIK signal at 10 μM, with an activity similar to that of established kinase inhibitors dasatinib and gefitinib. Nygren et al. carried out a screening using a cytotoxicity assay on human colon cancer cell lines (HCT116 and RKO) exposed to 10 μM of 1600 molecules with documented clinical use [64]. Sixty-eight compounds exerted activity on both cell lines, the highest frequency was obtained for known antineoplastic agents followed by anti-parasitic drugs; a distinct cluster containing anthelmintic benzimidazole compounds, including ABZ and FBZ, was observed. Mebendazole reduced the surviving fraction of cancer cells in a greater extent compared to ABZ and FBZ. Mebendazole was also found to be a potent inhibitor of several protein kinases including BCR–ABL and BRAF in the nanomolar range of concentration. Validation experiments were performed using human colon cancer cell lines HT29, HCT-8, and SW626 and non-malignant epithelial, renal, and hepatocyte cell lines. All five colon cancer cell lines showed an IC_50_ < 5 μM and three of them had an IC_50_ < 1 μM, whereas the drug was largely inactive in the non-malignant lines, thus indicating a potentially good therapeutic index.

### 4.7. Induction of Antitumor Immune Response

According to the binary polarization concept, macrophages are divided in two subtypes: classically activated (M1) have phagocytic and antigen presenting activity, produce Th-1 activating cytokines, and are therefore mediators of anti-tumoral response, while alternatively activated (M2) stimulate tumor progression promoting angiogenesis, matrix remodeling and immunotolerance [65]. Blom et al. [66] analyzed gene-expression profiles induced by MBZ in breast cancer MCF7 and promyelocytic leukemia HL60 cells, revealing that several of the most upregulated genes were related to monocyte/macrophage M1 phenotype activation. This finding was validated using THP-1 (a spontaneously immortalized monocyte-like cell line). Mebendazole resulted in strong upregulation of pro-inflammatory M1-phenotype genes encoding cytokines (such as *TNF*, *IL8* and *IL6*) surface markers (CD80 and CD 86) and T-cell-attracting chemokines, whereas no upregulation was observed for M2 markers. Mebendazole exposure induced IL-1β secretion, while no effect on IL-1β release was observed in response to other benzimidazoles or vincristine. In a co-culture model with differentiated THP-1 macrophages and HT29 colon cancer cells, MBZ activated a clear tumor suppressive effect. The immune effects of MBZ was further investigated by the same group in a co-culture of peripheral blood mononuclear cells (PBMCs), cancer cells, and either human fibroblasts or HUVEC cells [67]. Mebendazole (0.3–10 μM) increased release of pro-inflammatory cytokines, reduced levels of VEGF and VCAM-1, and potentiated killing of A549 NSCLC cells mediated by CD3/IL2 activated PBMCs. The effect seemed to be dependent on the presence of monocytes and macrophages since removal of CD14 expressing cells diminished the anti-tumor activity.

Finally, the role of protein kinase DYRK1B (dual specificity tyrosine-phosphorylation-regulated kinase 1B) as a mediator of the immune-modulating activity of MBZ was explored in a recent study by the same group [68]. Mebendazole revealed to be a potent inhibitor of DYRK1B (IC_50_ 360 nM, Kd 7 nM). As described before, MBZ was able to induce pro-inflammatory M1-type cytokines release in both THP-1 monocytes and THP-1 cells differentiated into macrophages. The DYRK1B inhibitor AZ191 induced M1 polarization only in macrophages, confirming that the inhibition of this kinase can partly recapitulate immune responses induced by MBZ.

## 5. Clinical Evidence of Anticancer Activity

To date, no results of clinical trials investigating MBZ as a cancer treatment are available, although two case reports have been published. In 2011, Dobrosotskaya et al. [69] reported the case of a 48-year-old man affected by metastatic adrenocortical carcinoma with progressive disease (right adrenal gland and multiple liver metastases) after repeated surgeries and radiation treatments for oligo-recurrence and systemic therapy with mitotane, 5-fluorouracil, streptozotocin, and bevacizumab. His treating physicians agreed to prescribe MBZ 100 mg b.i.d. following patient’s request: liver metastases initially regressed and subsequently remained stable for 19 months. The patient did not experience any significant adverse effect and described an improvement in quality of life. After 24 months of MBZ treatment, the disease progressed, even after the addition of everolimus. In 2013, Nygren and Larsson [70] published the case of a 74-year-old patient suffering from metastatic colon cancer in progression at multiple sites (lungs, abdominal lymph nodes, and liver) following two lines of chemotherapy with capecitabine, oxaliplatin, and bevacizumab and then capecitabine and irinotecan. Mebendazole was started at a dose of 100 mg b.i.d. and, after six weeks of monotherapy, a CT scan showed near complete remission of the metastases in the lungs and lymph nodes and a good partial remission in the liver. The patient experienced no adverse effects, but treatment was temporarily stopped for a transient elevation of liver enzymes and then reintroduced at half the dose. The disease was stable at the subsequent CT scan, confirming the response observed earlier.

Six active and/or recruiting clinical trials investigating the anticancer effect of MBZ, alone or in combination with other drugs, are currently registered at clinicaltrials.gov and are listed in Table 2.

## 6. Conclusions

The effectiveness of anticancer treatments is limited by refractory cell clones that are responsible for tumor progression. Intrinsic or acquired resistance is mediated by many molecular mechanisms including unregulated activation of pro-survival pathways and DNA repair enzymes, mutation or inactivation of tumor suppressors like p53, high levels of detoxifying proteins and transporter pumps mediating drug efflux, and immunotolerance and abnormal angiogenesis. Although resistant clones, including so called “cancer stem cells”, represent one of the main pitfalls of cancer treatment, there are currently no approved drugs specifically targeting this cell population. As one of the main limitations of drug development is its elevated cost, repurposing of already approved drugs is emerging as a promising mean to reduce the economic burden of drug research. This paper follows a comprehensive review, published in 2014 by Pantziarka et al. [71], and integrates it with new data obtained since then. Mebendazole was chosen as a candidate for antineoplastic treatment as it meets many of the characteristics desirable for a repurposed drug. Its favorable safety profile has been proven by decades of use as an anthelmintic agent, also at high dosages. Although its oral bioavailability is poor, MBZ reaches plasmatic and tissue concentration in the range of those that demonstrated antineoplastic activity in vitro [21,22,23,24,25] and is also able to cross the BBB [27]. Results of studies in vitro and in vivo supporting anticancer activity of MBZ are summarized in Table 3 and in Table 4. Mebendazole acts as an anti-tubulin agent, demonstrating inhibition of tubulin polymerization in several cancer cell lines, and has, therefore, also been proposed as a substitute for vincristine in the treatment of brain tumors [31]. Several preclinical studies proved the efficacy of MBZ as an inhibitor of multiple processes accountable for tumor resistance and progression at nano- and micro-molar clinically reachable concentrations: unregulated angiogenesis [29,35,36,37], pro-survival pathways (such as SHH [39], XIAP [41], MAPK/ERK [43], and c-MYB [45]), protein kinases activation and expression (including VEGFR2 [35], BRAF [43,62], MEK [43], BCR–ABL [64]), matrix metalloproteinase 2 [32] and multi-drug resistance protein transporters P-gp and MRP1 [47]. In vitro, MBZ was also able to stimulate antitumoral immune response by polarization of macrophages towards M1 tumor-suppressive phenotype [66,67,68]. Mebendazole demonstrated synergy with different chemotherapeutic agents, allowing to overcome chemoresistance to cisplatin [56], TMZ [53], and anti-HER2 conjugates with anthracycline or gemcitabine [52]. Mebendazole also exhibited potent activity as a radiosensitizer by enhancing radiation-induced DNA damage in cancer cells [50,51,55]. Induction of apoptosis through both the extrinsic and the intrinsic pathways was detected in vitro and in vivo in numerous cancer cell lines after MBZ treatment at concentrations achievable with the approved dosages [29,41,42,56,57,58,59]. Activity in vivo was tested in many murine models at oral dosages in the range of 25–100 mg/kg daily, that when converted in human equivalent doses based on body surface area [72] correspond to 2.03–8.13 mg/kg (142–569 mg daily for a patient weighting 70 kg), which is well below the currently approved dosages for anthelmintic therapy. In vivo, MBZ treatment as a single agent or in combination with chemotherapy led to reduction or complete arrest of tumor growth [29,35,36,41,43,45,57], marked decrease of metastatic spread [29,57], angiogenesis inhibition [29,35], and improvement of survival [27,30,31,35,39,43,45]. Two case reports [69,70] regarding the effective use of MBZ as an anticancer agent in metastatic patients support the feasibility of its application in a clinical setting. Other important features of MBZ are its ease of administration and its very low price in the majority of countries worldwide (with the remarkable exception of USA), allowing its diffusion also in low-income countries.

Although MBZ possesses many characteristics attractive for drug repurposing, there are still some possible drawbacks to be cleared. Targeting resistant clones and cancer stem cells is a challenging task, as CSC markers (including CD133, CD44, and ALDH) are not exclusively expressed by these cells’ sub-populations, and wide phenotype variability exists among CSCs from different patients and also within the same tumor. Several trials testing target-therapies inhibiting a single pathway led to dismal results, likely due to the over-activation of many survival mechanism characterizing these cells [73]. The ability of MBZ to inhibit different pathways and processes involved in tumor survival and progression might overcome this limit, and partly explains the effects on CSCs. On the other hand, its wide range activity could also affect stemness-associated factors shared between CSCs and normal stem cells. Moreover, this compound causes DNA damage and in vivo studies revealed its genotoxic and teratogenic effect, leading to skeletal abnormalities and malformations in rats and mice [25] and to malformation of the retinal layers in a zebrafish model [74]; for these reasons, MBZ is contraindicated during pregnancy. Mebendazole tolerability is excellent at the usual low-dose regimens and severe side effects have been rarely reported also during prolonged high-dose protocols, but the safety of its administration for a protracted time and in combination with approved antineoplastic treatments and in the oncology setting still have to be confirmed. Mebendazole has poor bioavailability with great inter-individual variability of pharmacokinetics [19,20,21,22,23,24,25] and, for some kind of tumors, its use as a single agent could require dosages near the maximum tolerated dose. In our opinion, MBZ should therefore be tested at relatively low doses as an adjuvant of already approved therapies, as it demonstrated efficacy as a sensitizer to chemotherapy and radiotherapy also at nano-molar concentrations. The ideal timing of MBZ use also has to be cleared: repurposed drugs in oncology are frequently tested only after failure of all the approved lines of treatment, but a greater effect could be expected in early phases of the disease, when CSCs are less numerous and developed less pro-survival adaptations. In the era of target therapy and immunotherapy, the development of a cheap broad-spectrum agent might sound counterintuitive. Nevertheless, the promises of precision medicine and “magic bullets” have largely been broken; despite having a clear advantage in tumors which are strongly dependent on specific driving pathways, these drugs are subject to the onset of resistance and the benefit is limited to a small percentage of patients [75]. In opposition to this paradigm, the concept of metronomic therapy gained momentum as a means to affect several processes involved in cancer progression by administration of continuous low-dose chemotherapy [76]. Likewise, repurposing of “dirty” drugs such as MBZ, acting over a wide range of pro-tumoral mechanisms, could help to overcome precision therapy limits and synergize with approved agents. Mebendazole also shares some effects with new generation anticancer agents, as it is able to inhibit several kinases [43,62,64] and stimulate antitumoral immune response [66,67,68]. Mebendazole’s low cost is a double-edged sword, as the lack of expected profit might discourage funding from pharmaceutical companies seeking a return on investments [77]. Academic and not-for-profit organizations could play a central role in supporting research in this field and spread the knowledge obtained to increase interest in clinicians: an example is the ReDO (Repurposing Drugs in Oncology) project [78], an international collaboration with the objective to gather and disseminate new evidence and accelerate the process of drug repurposing. Several papers have demonstrated the anti-cancer activity of other already approved anthelmintics including albendazole, flubendazole, and fenbendazole. These data confirm a certain class effect of benzimidazoles, also highlighted in drug screenings [58,64]. In our opinion, while all of these compounds are valid candidates for drug repurposing, MBZ holds some advantages over other benzimidazoles. Flubendazole and fenbendazole at the moment are approved only for veterinary use and literature regarding their repurpose against cancer is more limited. Albendazole is approved for human use and characterized by a good toxicity profile, but its safety for long-term use is unknown as it is prescribed at lower doses (10 mg/kg) for up to three monthly courses [79] and higher dosages, although fairly tolerated, were limited by neutropenia [80]. Evidence for anti-neoplastic effectiveness of ABZ is convincing, but in comparison to MBZ fewer studies have been published and the only three papers we could find in which the anticancer effect of both drugs has been tested revealed a less evident activity of ABZ in vitro and in vivo [30,58,64]. In conclusion, the data summarized in this review confirm mebendazole as an ideal candidate for drug repurposing, warranting further investigations in clinical trials to confirm its safety in the oncology setting and activity as an anticancer treatment.

## Figures and Tables

**Table 1:** Pharmacokinetic studies in patients treated for hydatid disease. Cmax = peak serum concentration AUC = area under the curve T1/2 = elimination half-life.

Dosage	C_max_ and AUC	Half-Life of Elimination	Tissue Concentrations
10 mg/kg, single dose or chronic administration, 12 patients treated for cystic hydatid disease [21]	Cmax 17.5 to 500 ng/mL (0.06–1.69 µM, mean 0.24 µM) after a single dose. In chronic therapy mean C_max_ 0.47 µM and AUC five times higher than after single dose	T_1/2_ 2.8–9.0 h, time to peak plasma concentration 1.5–7.25 h	Concentrations of MBZ found in the tissue and cyst material collected from two patients during surgery ranged from 59.5 to 206.6 ng/g wet weight
1–12 g/day, 17 patients treated for hydatid cysts and 5 volunteers [22]	C_max_ 0.03–1.64 µM	T_1/2_ 3.3–11.5 h	-
1000 mg single dose, 8 healthy volunteers [23]	mean Cmax 0.11 µM, mean AUC 207.2 µg·h/L	T_1/2_ mean 7.4 h	-
1.5 g single dose or repeated 1 g administrations [24]	Cmax 0.017–0.134 µM after single dose and up to 0.5 µM after repeated administrations	-	-

**Table 2:** Ongoing studies registered at Clinicaltrials.Gov investigating MBZ as a repurposed drug in oncology. MTD = maximum tolerated dose.

Phase	Condition	Intervention	Institution
Phase 1	Newly diagnosed high-grade glioma	Standard of care (surgery and radio-chemotherapy) followed by MBZ (MTD to define) + adjuvant sequential TMZ.	Sidney Kimmel Comprehensive Cancer Center at Johns Hopkins
Phase 1	Pediatric patients affected by medulloblastoma or high-grade glioma in progression after standard therapies	MBZ alone (MTD to define)	Sidney Kimmel Comprehensive Cancer Center at Johns Hopkins
Phase 1–2	Pediatric patients affected by low- or high-grade glioma	MBZ 50–200 mg/kg/day (MTD to define) in combination with vincristine, carboplatin, and temozolomide (low grade) or bevacizumab and irinotecan (high-grade glioma)	Cohen Children’s Medical Center of New York
Phase 2	Advanced or metastatic gastrointestinal cancer or cancer of unknown origin	MBZ alone, dose escalation (50–4000 mg), and pharmacokinetic analysis	Uppsala University
Phase 2	Metastatic or advanced cancer (different organs and histology)	MBZ 100 mg b.i.d. + metformin up to 1000 mg b.i.d. + doxycycline 100 mg/die + atorvastatin up to 80 mg/die; “real world setting”, with or without concomitant standard of treatment	Care Oncology Clinic, London
Phase 2	Stage IV colorectal cancer	MBZ (dose not specified) concomitant with adjuvant FOLFOX + bevacizumab	Tanta University

**Table 3:** Studies reporting MBZ anticancer activity in vitro and its mechanisms of action. IC_50_ = half maximal inhibitory concentration; EC_50_ = concentration required to achieve a half-maximal effect.

Author Year	Cell Line	Ic_50_ Antiproliferative	Biological Effect
Mukhopadhyay T et al. 2002 [33]	Human Non-Small Cell Lung Cancer (NSCLC): A549, H1299, H460. Human breast, ovary, and colon carcinoma and osteosarcoma	NSCLC cell lines: ~0.16 µM. Other cell lines: 0.1–0.8 μM.	Not specified. Growth inhibition of about five-fold after exposure of H460 and A549 cells to 0.165 μM for 5 days. No effect on HUVECs and normal fibroblasts also at 1 μM.
Sasaki J et al. 2002 [29]	Three human NSCLC cell lines	A549 0.417 µM, H1299 0.260 µM, H460 0.203 µM	0.5 µM tubulin depolymerization. Induction of p53 and p21 expression after 24 h, induction of apoptosis after 48 h in 35% of H460 cells and 15% of A549 cells.
Martarelli D et al. 2008 [57]	Human adrenocortical cancer H295R and SW-13	H295R 0.23 μM, SW-13 0.27 μM	Cell invasion inhibition (0.085 μM). Cytochrome c and caspase-9 and 3 mediated apoptosis.
Doudican NA et al. 2008 [41]	Human melanoma M-14 and A-375	Not specified	Decrease in XIAP levels, increase in apoptosis markers (cleaved PARP and caspase 9) at 0.5 μM.
Bai RY et al. 2011 [30]	A panel of 10 glioblastoma cell lines.	Between 0.11 and 0.31 μM	Inhibition of tubulin polymerization in 060919 cells at 0.1 µM for 72 h.
Doudican N et al. 2013 [58]	Human melanoma SK-Mel-19 and M-14	SK-Mel-19 0.32 µM, M14 0.30 µM.	Apoptosis induction at 1 µM for 24 h in 25% of M-14 cells and 31% of SK-Mel-19. At 0.5 µM only 26% of SK-Mel-19 cells maintained proliferative capacity.
Nygren P et al. 2013 [64]	Human colon cancer cell lines HT29, HCT-8 and SW626, HCT 116 and RKO	Less than 5 μM for all the lines tested and <1 μM for 3 lines	Inhibition of several kinases (including BCR–ABL and BRAF) in the nanomolar range.
Coyne CP et al. 2014 [52]	Human mammary adenocarcinoma SKBr-3	About 0.35 μM at 96h and 0.25μM at 182 h. IC_80_ at 182 h ~0.30 μM	Survival fraction reduced to 36.9–9.2% after exposure to 0.2–2.5 μM for 96–182 h. Synergy with anti-HER2 conjugates with anthracyclines or gemcitabine: 0.15 μM MBZ ↓ survival fraction from 48.7% to 7.7% at chemotherapeutic-equivalent concentrations of 10^−8^ M and from 79.5% to 8.7% at 10^−10^ M.
Larsen AR et al. 2015 [39]	DAOY human medulloblastoma	Not specified	Sonic hedgehog (SHH) pathway inhibition: inhibition o.d. SMO mutant proteins and reduction in GLI1 expression (0.1–1 μM, IC_50_ 0.516 μM). Inhibition of cell proliferation (0.1 μM) and primary cilium assembly, induction of apoptosis (1 μM).
Bai RY et al. 2015 [35]	A panel of 8 medulloblastoma cell lines	Between 0.13 and 1 μM after 72 h	Inhibition of VEGFR2 autophosphorylation, at 1–10 μM in cultured HUVECs and with an IC50 of 4.3 μM in a cell-free kinase assay.
*Pinto* LC et al. 2015 [32]	Human gastric cancer ACP-02, ACP-03 and AGP-01 (malignant ascites)	ACP-02 0.39 μM, AGP-01 0.59 μM, ACP-03 1.25 µM	Disruption of microtubules, inhibition of invasion and migration and of MMP-2 activity.
Williamson T et al. 2016 [36]	Colo-rectal carcinoma cell lines DLD-1, HCT-116, HT29, and SW480	DLD-1 0.28 μM, HCT-116 0.25 μM, HT29 0.20 μM, and SW480 0.81 μM	Not specified.
Simbulan-Rosenthal CM et al. 2017 [43]	Patient-derived melanoma NRAS mutated (BAK and BUL) and BRAF mutated (STU)	Not specified	Inhibition of several kinases, including BRAF wild type and BRAFV600E (with a Kd of 210 and 230 nM) and MEK. Inhibition of MAPK/ERK pathway, induction of apoptosis, synergy with trametinib
Zhang F et al. 2017 [56]	Human head and neck squamous cell carcinoma CAL27 and SCC15	CAL27 1.28 and SCC15 2.64 μM	Apoptosis induction as a single drug. Strong synergistic effect with cisplatin. Increase in CAL27 and inhibition in SCC15 cells of proliferation related pathways
De Witt M et al. 2017 [31]	GL261 murine glioma	Cell viability suppression 160 nM	EC_50_ for microtubule depolymerization 132 nM, mitotic arrest induction 192 nM
Pinto LC et al. 2017 [47]	AGP-01 intestinal type adenocarcinoma	Not specified	Inhibition of P-gp and MRP1 at 1.0 μM for 24 h. Inhibition of MATE1 at 0.1–1.0 μM
Blom K et al. 2017 [66]	THP-1 monocyte and HT29 colon cancer co-culture	Not specified	1–10 μM for 6 h increased release of pro-inflammatory M1 cytokines (such as IL-1β, TNF, IL8, and IL6) and surface markers (CD80 and CD 86), induction of antitumor response in co-culture. Induction of IL-1β secretion in presence (1 μM) or in absence (10 μM) of LPS. Induction of tumor suppressive effect in co-cultures
Markowitz D et al. 2017 [50]	Human GBM14 glioblastoma Murine GL261 glioma	Not specified	Radiosensitization with an EC_50_ of 35 nM. Cytoplasmic sequestration of DDRp Chk2 (EC_50_ 31 nM) and Nbs1 (EC_50_ 25 nM)
Walf-Vorderwülbecke V et al. 2018 [45]	Eight different Acute Myeloid Leukemia cell lines	IC50s for cell viability between 0.07 and 0.26 µM	Degradation of c-MYB and inhibition of its expression. Reduction in colony formation (>80% after exposure of THP1 AML cells for 16 h at 10 µM)
Rubin J et al. 2018 [67]	Co-culture of PBMCs, A549 cells and human fibroblasts or HUVEC cells	Not specified	0.3–10 μM increased release of pro-inflammatory cytokines, reduced levels of VEGF and VCAM-1, potentiated killing of A549 NSCLC cells mediated by CD3/IL2 activated PBMCs
Skibinski CG et al. 2018 [55]	Seven meningioma cell lines	IC50s for cell viability after 72 h 0.26–0.42 μM	Reduced clonogenic activity, induced cytotoxicity, increased levels of cleaved caspase-3 and PARP and reduced colony formation
Kralova V et al. 2018 [48]	PE/CA-PJ15 and H376 oral SCC; DOK premalignant oral keratinocytes	Not specified	PE/CA-PJ15 and H376: 0.1–0.25 μM MBZ or FBZ inhibition of kinases (FAK) and GTPases (Rho-A, Rac1); dose dependent migration inhibition (0.1–5 μM) DOK: TGF-β induced N-cadherin inhibited at 0.05–0.2 μM for 48 h
Zhang L et al. 2019 [51]	SUM159PT and MDA-MB-231 TNBC	0.35 µM in monolayers and 0.4 µM in mammospheres after 72 h.	0.5 µM arrest in the G2/M phase; significant radiosensitizing effect at all radiation doses tested (1–8 Gy). Mebendazole at 0.35 and 0.7 µM dose-dependent decrease of ALDH1 positive CSCs; Hedgehog pathway inhibition. ↑ fraction of apoptotic cells, ↑ DNA DSBs
Sung SJ et al. 2019 [37]	HUVECs	IC_50s_ for cell proliferation after 48 h 0.7–2.5 μM	Inhibition of VEGF or bFGF induced migration (IC_50_ 0.7–0.9 μM) and tube formation (IC50 0.8–1.5 μM); ↑ p53 level up to 2.9 fold Dose and time-dependent apoptosis in up to 34% of cells at 72 h
Blom K et al. 2019 [68]	THP-1 monocytes and macrophages.	Not specified	DYRK1B inhibition IC_50_ of 360 nM and kD of 7 nM. 10 µM for 24 h ↑ M1 marker CD80 and ↓ M2 marker CD163
Pinto LC et al. 2019 [59]	AGP01 gastric cancer	Not specified	0.5–1 μM ↑ caspase 3 and 7 activity, ↓ C-MYC mRNA and C-MY. Cell cycle arrest in G0/G1 and G2/M phases at 0.5 μM and 1.0 μM. Apoptosis induction 68% (0.5 μM) and 74% (1 μM) of cells at 72 h

**Table 4:** Studies reporting MBZ anticancer activity in vivo and its mechanisms of action. Abbreviations: e.o.d = every other day; I.p. = intra-peritoneally.

Author and Year	CELL LINES TESTED	DOSE	BIOLOGICAL EFFECT IN VIVO	ANTITITUMOR EFFECT
Mukhopadhyay T et al. 2002 [33]	H460 and A549 human NSCLC. K1735 murine melanoma.	0.4–0.8–1 mg/mouse/e.o.d. (oral)	Angiogenesis inhibition Metastatic spread inhibition	H460: tumor growth inhibition of 30% (0.4 mg) and 80% (0.8 mg) and almost complete arrest of growth (1 mg/mice/e.o.d.) A549: 80% reduction of metastases number in lungs (1 mg/mouse/e.o.d.) K1735 allograft: 1 mg growth inhibition of ~70%.
Martarelli D et al. 2008 [57]	H295R and SW-13 human adrenocortical cancer	1 or 2 mg/mice/day (oral)	Apoptosis induction Invasion inhibition Metastatic spread inhibition	H295R: about 50% (1 mg) and 60% (2 mg) tumor volume reduction SW-13: about 70% (1 mg) and 60% (2 mg) tumor volume reduction and 50% (1 mg) and 75% (2 mg) reduction of lung metastases number
Bai RY et al. 2011 [30]	GL261 murine glioma and 060,919 human GBM	50 mg/kg (oral)	Not specified	Survival increase in GL261: 29 d CTRL vs. 41 d TMZ vs. 49 d MBZ vs. 50 d TMZ + MBZ vs 36 d ABZ 50 mg/kg vs 39 d ABZ 150 mg/kg Survival increase in 060919 xenograft: 48 d CTRL versus 65 d MBZ vs 43 d ABZ 150 mg/kg
Doudican NA et al. 2013 [41]	M-14 human melanoma	1 or 2 mg/mouse/day (oral by gavage)	XIAP inhibition Apoptosis induction	Tumor growth inhibition of 83% (1 mg) and 77% (2 mg)
Larsen AR et al. 2015 [39]	DAOY human medulloblastoma	25–50 mg/kg (oral)	Sonic Hedgehog pathway inhibition	Survival increase: 75 d control group (CTRL) versus 94 d MBZ 25 mg/kg versus 113 d MBZ 50 mg/kg
Bai RY et al. 2015 [35]	D425 human medulloblastoma. Murine parental or SMO-D477G mutated medulloblastoma.	50 mg/kg/day (oral in food)	Angiogenesis inhibition	Survival increase in murine medulloblastoma: 150% increase in the parental line and 100% in SMO-D477G mutated allograft; growth inhibition in both models. Survival increase in D425 xenograft: 125% increase in survival versus CTRL; tumor burden reduction.
Bai RY et al. 2015 [27]	GL261 murine glioma D425 human medulloblastoma	50 mg/kg of polymorph A, B or C MBZ (oral by gavage)	Not specified	Survival increase, enhanced by elacridar (ELD) GL261:29 d CTRL vs. 34 d ELD vs. 53 d MBZ vs. 92.5 d MBZ + ELD (for 7 days) vs. 110.5 d MBZ + ELD (for 14 days) D425: 24 d CTRL vs. 33 d ELD vs. 52 d MBZ vs. 77 d MBZ + ELD (for 7 days)
Williamson T et al. 2016 [36]	HT29 or SW480 human colorectal cancer APCmin/+ model	50 mg/kg or 35 mg/kg (oral by gavage)	Inhibition of several pathways (MYC, COX2 and Bcl-2) and cytokines. Angiogenesis inhibition.	Tumor volume and weight reduction: respectively 62% and 65% in HT29 and 67% and 59% in SW480 (50 mg/kg) APCmin/+ chemoprevention model: reduction of tumor numbers 56% as a single agent (35 mg/kg) and up to 90% in combination with sulindac
Simbulan-Rosenthal CM et al. 2017 [43]	BAK human melanoma	40 mg/kg (oral by gavage)	MEK1/2 and ERK1/2 inhibition	MBZ or trametinib (1 or 3 mg/kg) showed no growth inhibition as single agents, in combination 50% volume reduction and increased survival
Zhang et al. 2017 [56]	CAL27 human head and neck squamous cell carcinoma	7.5 mg/kg i.p. e.o.d	Cell differentiation	Slight volume increased, induction of cell differentiation (extensive keratinization, diminished expression of proliferation markers and up-regulated expression of differentiation markers).
De Witt M et al. 2017 [31]	GL261 murine glioma	50–100 mg/kg of polymorph C MBZ (oral)	Not specified	Survival increase: 10 d CTRL vs 11 d voncristine vs 17 d MBZ 50 mg/kg vs. 19 d MBZ 100 mg/kg
Walf-Vorderwülbecke V et al. 2018 [45]	THP1 human acute myeloid leukemia	200 mg/kg of diet (oral, mixed in food)	c-MYB degradation	Growth inhibition and survival increase (~ 65 days vs. ~40 days in CTRL group)
Skibinski CG et al. 2018 [55]	KT21MG1 human meningioma	50 mg/kg/day in high fat diet	Apoptosis induction, angiogenesis inhibition	KT21MG1 intercranial xenograft: median survival 19 d in CTRL group, 30 d MBZ 33.5 d RT (12 Gy) and 39 d RT + MBZ
Zhang L et al. 2019 [51]	SUM159PT human TNBC	10 or 20 mg/kg 5 days/week i.p.	Radiosensitization	MBZ alone modest effect, IR 10 Gy evident growth delay potentiated by MBZ 20 mg/kg
